# Involvement of Endogenous Opioid System in Swim Stress-Induced Pain Modulation During the Interphase of the Formalin Test

**DOI:** 10.32598/bcn.9.10.220

**Published:** 2019-07-01

**Authors:** Ali Reza Moslem, Bahareh Amin, Nima Heidari-Oranjaghi, Hassan Azhdari-Zarmehri

**Affiliations:** 1. Department of Anesthesiology, School of Medicine, Sabzevar University of Medical Sciences, Sabzevar, Iran.; 2. Cellular and Molecular Research Center, Department of Physiology and Pharmacology, School of Medicine, Sabzevar University of Medical Sciences, Sabzevar, Iran.; 3. Department of Physiology and Pharmacology, School of Medicine, Zanjan University of Medical Sciences, Zanjan, Iran.; 4. Neurosciences Research Center, Torbat Heydariyeh University of Medical Sciences, Torbat Heydariyeh, Iran.; 5. Department of Physiology, School of Paramedical Sciences, Torbat Heydariyeh University of Medical Sciences, Torbat Heydariyeh, Iran.

**Keywords:** Swim stress, Rat, Formalin test, Naloxone

## Abstract

**Introduction:**

Some evidence demonstrates endogenous inhibitory pathways of pain involved in the interphase (phase between early and later phase) of the formalin test. We previously showed that swimming stress modulates the pain-related behaviors during the interphase of the formalin test. In this study, we evaluated the role of the endogenous opioid system in modulating nociceptive responses of the formalin test.

**Methods:**

Swim stress was performed in different heights of water (5, 25, 50 cm) in a swimming tank. The mean nociceptive scores were measured during phase 1 (1–7 min), interphase (8–14 min), and phase 2 (15–90 min) of the formalin test. Opioid receptor antagonist, naloxone (3 mg/kg; IP) was injected immediately before swim stress.

**Results:**

Swim stress attenuated nociceptive behaviors in the first phase and increased the duration of interphase in the formalin test in a water-height-dependent manner, compared to the control group. Naloxone significantly increased nociceptive behaviors in the first phase, interphase, and the second phase of the formalin test, compared to the control group.

**Conclusion:**

Stress could affect the nociceptive response. Swim stress in different heights of water could have different effects on the nociception in different phases of the formalin test. In addition, the involvement of the endogenous opioid system is further demonstrated in the swim stress-induced modulation of pain behaviors in phase 1, phase 2, as well as interphase of formalin test in rats.

## Highlights

- Swim stress attenuated nociceptive behaviors in the first phase and increased the duration of interphase in the formalin test in a water-height-dependent manner.
- Naloxone increased nociceptive behaviors in the first phase, interphase, and phase 2 of the formalin test.
- Involvement of endogenous opioid system demonstrated in the swim-stress-induced modulation of pain behaviors in phase 1, phase 2, and interphase of formalin test.

## Plain Language Summary

Formalin test is commonly used as an animal model of tonic pain to evaluate the analgesic effects of drugs. Stress has an important role in the modulation of interphase. The interphase period in the formalin test may be an active process due to the activity of endogenous pain-suppressing mechanisms. Swim-stress-induced analgesia appears to be mediated through opioid and non-opioid mechanisms. Based on the previous studies, swim stress prolonged interphase or delayed the start of phase 2 in the formalin test. In addition, some studies have shown that naloxone can modulate only the interphase, while testosterone involves in phases 1 and 2 without affecting the interphase. We hypothesized that swim stress could modulate the nociceptive behaviors induced by formalin in phase 1, interphase, and phase 2, through interacting with the endogenous opioid system.

## 1. Introduction

Formalin test is commonly used as an animal model of tonic pain and even sometimes as a chronic pain model to evaluate potential analgesic effects of drugs (Abbott, Franklin, & Westbrook, 1995; Dubuisson & Dennis, 1977). Injection of low concentration of formalin into the hind paw of animals induces a series of nociceptive behaviors which could last for more than one hour (Abbott et al., 1995; Dubuisson & Dennis, 1977; Sofiabadi et al., 2011). Stress has an essential role in the modulation of interphase, this period is much overlooked since it is probably considered a phase of inactivity (Gaumond, Arsenault, & Marchand, 2002; Henry, Yashpal, Pitcher, & Coderre, 1999).

Some data demonstrate that interphase period in the formalin test may be an active process, comprising endogenous pain-suppressing mechanisms (Gaumond et al., 2002; Henry et al., 1999; Franklin & Abbott, 1993). Pentobarbital, diazepam, and ethanol inhibit the alleviation of pain during the interphase through activating Gamma-Aminobutyric Acid (GABA) receptors (Franklin & Abbott, 1993). Henry et al. (1999) reported that after two injections of formalin (with 20 min interval), a triphasic response was seen with another diminution of nociceptive scores, compared to a biphasic response in one injection of formalin.

Swim Stress-induced Analgesia (SSIA) can attenuate formalin-induced nociceptive responses. This form of analgesia appears to be mediated through opioid and non-opioid mechanisms (Lapo, Konarzewski, & Sadowski, 2003). Based on the previous studies, swim stress prolongs interphase or delay the start of the second phase in the formalin test. In addition, some studies have shown that naloxone can modulate only the interphase, while testosterone is involved in phases 1 and 2 without affecting the interphase (Gaumond, Arsenault, & Marchand, 2005; Gaumond, Spooner, & Marchand, 2007). We hypothesized that swim stress could modulate the nociceptive behaviors induced by formalin in phase 1, interphase, and phase 2, through interaction with the endogenous opioid system. To test this hypothesis, we assessed the effect of an opioid antagonist, naloxone, on the swim stress-induced pain modulation during phase 1, interphase, and phase 2 of the formalin test.

## 2. Methods

### 2.1. Study animals

All experiments were performed following the National Institutes of Health Guide for Care and Use of Laboratory Animals (NIH Publication No. 80-23, revised 1996), and approved by the Ethics Committee of Sabzevar University of Medical Sciences, Sabzevar, Iran. Maximum effort was made to minimize discomfort and number of study animals. Young male, Sprague-Dawley rats (weight: 80–120 g) were maintained in a temperature controlled room with a 12:12 h light-dark cycle with lights on from 7:00 to 19:00 and housed in groups by threes in a cage. Food and water were provided ad libitum.

Sixty-one animals were divided into eight groups (n=7–8); groups 1 and 2: animals were not exposed to swim stress (control) and those treated with naloxone, respectively; groups 3 and 4: Animals subjected to swim stress in 5-cm height water and those treated with naloxone; groups 5 and 6: animals subjected to swim stress in 25-cm height water and those treated with naloxone; groups 7 and 8: Animals subjected to swim stress in 50-cm height water and those treated with naloxone.

### 2.2. Swim stress-induced analgesia

To assess the effects of swim stress on the pain induced by formalin injection, the rats were acclimatized in the formalin test box for 30 min. Then they were subjected to forced swim stress for 3 min in a cylinder plastic tank (60 cm height and 50 cm diameter), filled with 20°C water with heights of 5, 25 or 50 cm. Animals in the control group did not receive any stress procedure. The water was clear, and 2 rats simultaneously were used for swim stress paradigm. After ending swimming sessions, each rat was carefully dried with a new towel and placed into formalin test box for 5 min to acclimate, and then formalin was injected into the plantar surface of the rat’s right hind paw.

### 2.3. Formalin Test

After swimming, the animals were taken back into the formalin test chamber to acclimatize. Formalin (50 Μl, 2%) was injected Subcutaneously (SC) into the plantar surface of the right hind paw with a 30-gauge needle. Animals were then immediately returned to the observation box to record their pain behaviors. A mirror was placed at a 45° angle beneath the box for accurate detection of pain behaviors without moving the box. Pain behaviors were scored as follows: 0. The injected paw was not favored; 1. The injected paw had little or no weight placed on; 2. The injected paw was elevated and not in contact with any surface; and 3. The injected paw was licked or bit.

Scores were continuously observed during the 90 min of the experiment. The scores were recorded in control rats as well as those who were put in 5, 25, and 50 cm heights of water in the swimming tank. In each group, the response of each rat during the first phase (1–7 min), interphase (8–14 min), and the second phase (15–90 min) was separately recorded (Azhdari-Zarmehri, Erami, Ghasemi, & Salmani, 2012).

### 2.4. Data analysis

The obtained data were expressed as Mean±SEM, and analyzed by 1-way Analysis of Variance (ANOVA) and t-test. The mean nociceptive scores in each phase I (1–7 min), interphase (8–14 min), phase 2A (15–60 min) and 2B (60–90 min) in the formalin test and in different heights of water were analyzed using ANOVA followed by Dunnett’s and or Tukey’s post hoc tests. P values less than 0.05 were considered as a significant difference (Azhdari Zarmehri et al., 2012).

## 3. Results

### 3.1. Effects of naloxone on the mean nociceptive scores of the formalin test

**Figure 1.**
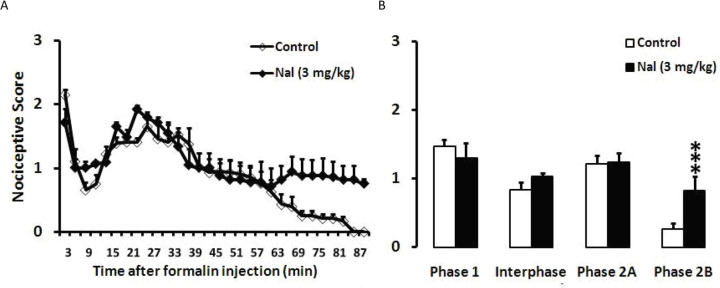
The effect of Naloxone (Nal) injection (3 mg/kg, IP) on the formalin-induced nociceptive scores A. Time scores of formalin-induced nociceptive behaviors were measured every 3 min during 90 min; B. Bar charts represent nociceptive scores of phase 1 (1–7 min), interphase (8–14 min), the first part (15–60 min) and the second part (61–90 min) of phase 2 in the formalin test, in the control and naloxone groups. ^***^P<0.001 compared to the control group; Values are presented as Mean±SEM.

In the control group with no swim stress, SC injection of formalin into the hind paw induced typical biphasic pain responses. Naloxone (3 mg/kg) was intraperitoneally administered before formalin injection. Naloxone produced a similar nociceptive behavior score during phase 1, interphase, and phase 2A, except for phase 2B of the formalin test (for phase 1: t (14)=0.84; P=0.417; for interphase: t (14)=1.375; P=0.194; for phase 2A: t (14)=0.069; P=0.946; and for phase 2B: t (14)=5.183; P=000). Based on the t-test analysis, naloxone significantly increased nociceptive behaviors during the end of phase 2 compared to the control (P<0.001) (Figure 1).

### 3.2. Effects of naloxone on the swim stress-induced pain modulation in the 5-cm height of water on mean nociceptive scores of the formalin test

**Figure 2.**
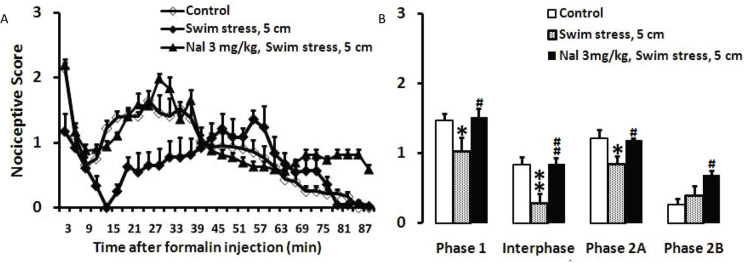
The effects of Naloxone on the formalin test after swim stress (5 cm) A. Time scores of formalin-induced nociceptive behaviors following swim stress were measured every 3 min for 90 min; B. Bar charts represent nociceptive scores of phase 1, interphase, first and second parts of phase 2 of the formalin test in the control, swim stress, and swim stress+naloxone-treated rats. Values are presented as Mean±SEM; The height of the water was 5 cm; ^*^P<0.05 and ^**^P<0.01 in comparison with the control group and ^#^ P<0.05 and ^##^ P<0.01 in comparison with the swim stress group.

Swim stress in the 5-cm height of water decreased mean nociceptive score in phase 1 (P<0.05), interphase (P<0.01), and the first part of phase 2 (P<0.05), but it did not affect the second part of phase 2. Naloxone (3 mg/kg; IP) was injected immediately before swimming and followed by formalin injection. As observed in Figure 2 A–B, the injected naloxone significantly prevented the antinociceptive responses of swimming after application of formalin. One-way ANOVA of data revealed a significant difference for the phase 1 (F_2, 19_=12.710, P=0.001), interphase (F_2, 19_=9.166, P=0.002), phase 2A (F_2, 19_=3.616, P=0.04), and phase 2B (F_2, 19_=4.525, P=0.025), (Figure 2).

### 3.3. Effects of naloxone on the swim stress-induced pain modulation in the formalin test in the 25-cm height of water on mean nociceptive scores of the formalin test

**Figure 3.**
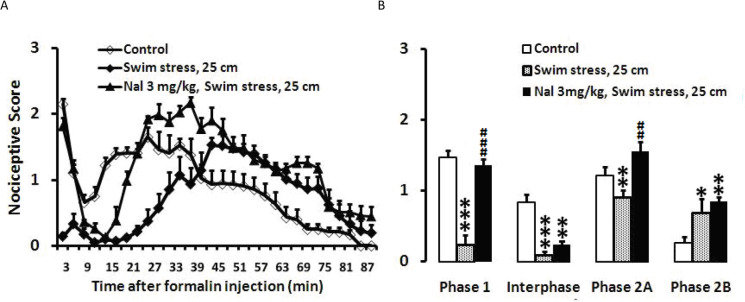
The effects of Naloxone on the formalin test after swim stress (25 cm) A. Time scores of formalin induced-nociceptive behaviors fo llowing swim stress were measured every 3 min for 90 min; B. Bar charts represent nociceptive scores of phase 1, interphase, the first and second parts of phase 2 in the formalin test in the control, swim stress, and swim stress+naloxone-treated rats. Values are presented as Mean±SEM; ^*^P<0.05; ^**^P<0.01 and ^***^P<0.001 in comparison with the control group and ^##^P<0.01 and ^###^ P<0.001 in comparison with the swim stress group; The height of water were 25 cm.

Swim stress in 25-cm height of water decreased nociceptive behaviors in phase 1 (P<0.001), interphase (P<0.001), and phase 2A (P<0.01), but had pronocicep Naloxone (3 mg/kg; IP) was injected immediately before swim stress, followed by formalin injection. The weighted pain scores were recorded at 3-min intervals during a 90 min period. Swim tank was filled with water up to a height of 25 cm. As observed in Figure 3A–B, the IP injection of naloxone prevented the antinociceptive responses of the swim in phase 1 (P<0.001) and phase 2A (P<0.01) as demonstrated in Figure 3B. One-way ANOVA of data revealed a significant difference for phase 1 (F_2, 19_ =48.838, P=0.000), interphase (F_2, 19_=15.760, P =0.000), phase 2A (F_2, 19_ =5.032, P=0.018), and phase 2B (F_2, 19_=4.872, P=0.020) (Figure 3).

### 3.4. Effects of naloxone on the swim stress-induced pain modulation in the 50-cm height of water on mean nociceptive scores of phase the formalin test

**Figure 4.**
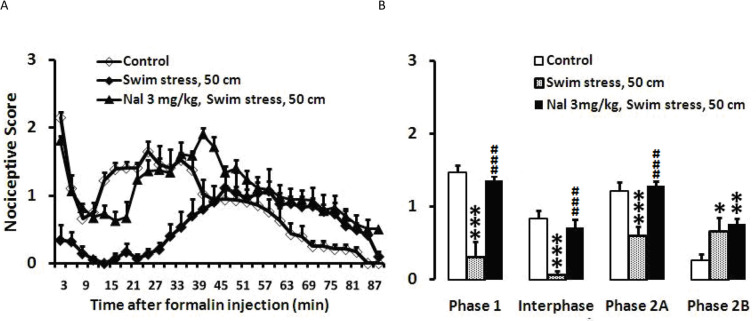
The effects of Naloxone on the formalin test after swim stress (50 cm) A. Time scores of formalin-induced nociceptive behaviors following swim stress were measured every 3 min for 90 min; B. Bar charts represent nociceptive scores of phase 1, interphase, first and second parts of phase 2 in the formalin test in the control, swim stress, and swim stress+naloxone-treated rats; Values are presented as Mean±SEM; ^*^ P<0.05; ^**^ P<0.01 and ^***^ P<0.001 in comparison to the control group and ^###^ P<0.001 in comparison with the swim stress group; The height of water were 25 cm.

Swim stress in 50-cm height of water decreased nociceptive behaviors in phase 1(P<0.001), interphase (P<0.001), and phase 2A (P<0.001), but had pronociceptive effect during the second part of phase 2 of formalin test (P<0.01). Naloxone (3 mg/kg; IP) was injected immediately before administration of swim stress, followed by formalin injection and recording the weighted pain scores at 3-min intervals during a 90 min period. As observed in Figure 4 A–B, naloxone significantly prevented the antinociceptive responses of swimming in phase 1 (P<0.001), interphase (P<0.001), and phase 2A (P<0.001) as demonstrated in Figure 4B, and had no effect on pronociceptive effect during second part of phase 2 of formalin test (for phase 1: F_2, 22_=31.334, P=0.000; for interphase: F_2, 22_=24.452, P=0.000; for phase 2A: F_2, 22_=16.150, P=0.000; and for phase 2B: F_2, 22_=3.453, P=0.050) (Figure 4).

## 4. Discussion

In the current study, we found that opioid receptor antagonist, i.e. naloxone, significantly increased nociceptive behaviors during the end of phase 2 of the formalin test in animals without swim stress compared to the control group. It seems that a powerful endogenous opioid inhibitory mechanism is responsible for nociceptive behavior termination at the end of the formalin test (Azhdari-Zarmehri, Mohammadzadeh, Feridoni, & Nazeri, 2014). Swim stress decreased the nociceptive behaviors in the first phase of the formalin test. Conversely, it prolonged interphase of the formalin test in comparison to the control ones in a water-height-dependent manner indicating different pain modulation during different phases of the formalin test and elucidated the impact of swim stress on the duration of interphase.

The interphase period has been long considered as an inactive phase, but some research studies suggest that active inhibitory mechanisms are involved in the modulation of pain during this period (Gaumond et al., 2002; Henry et al., 1999; Franklin & Abbott, 1993). Stress has been shown to activate multiple neural systems involved in pain sensation and modulation (Bodnar, Kelly, Brutus, & Glusman, 1980; Guillemin et al., 1977; Madden, Akil, Patrick, & Barchas, 1977). This endogenous pain inhibitory systems (Bodnar et al., 1980; Guillemin et al., 1977; Madden et al., 1977). In a stressful situation, the opioid and non-opioid forms of SIA are elicited in rodents (Bodnar et al., 1980; Madden et al., 1977). It has been reported that antagonizing the endogenous opioid system with naloxone or naltrexone attenuates the nociceptive behavioral responses following exposure to stress, supporting the role of the endogenous opioid system in SIA (Amit & Galina, 1986). Systemic or intracerebroventricular injection of μ-, κ-, or δ-opioid receptors antagonists prevents the SIA or fear-conditioned analgesia in rats (Akil, Young, Walker, & Watson, 1986; Butler & Finn, 2009; Fanselow, Calcagnetti, & Helmstetter, 1989). The characteristics of a stressor such as duration, intensity, and temporal aspects, affect the induced analgesic response (Watkins & Mayer, 1986; Amit & Galina, 1986). SSIA can attenuate formalin-induced nociceptive responses (Hopkins, Spinella, Pavlovic, & Bodnar, 1998; Altier & Stewart, 1999). This form of analgesia is considered to be mediated through opioid and non-opioid mechanisms (Lapo et al., 2003; Oliverio & Castellano, 1982; Mogil et al., 1993).

Mogil et al. (1993) showed that swimming stress in 20°C water could induce analgesia that is sensitive to naloxone in male rats. In contrast, antagonizing opioid receptors were not effective in inhibiting SSIA in female rats, suggesting that other mechanisms depending on the sex hormones might be involved. According to our previous study, exposure to restraint stress as well as swim stress significantly reduces the formalin-induced nociceptive behaviors in rats. An orexin receptor type 1 antagonist entirely reverses this antinociceptive effect produced by either restraint stress or swim stress on interphase. The opioid receptor antagonist, naloxone, does not reverse the observed antinociceptive effect with such forms of stress. Interphase is considered an inactive phase, but recent evidence shows the involvement of active mechanisms in this quiescent phase (Henry et al., 1999).

Based on previous reports, pentobarbital, diazepam, and ethanol attenuate nociceptive behaviors in the interphase of the formalin test, which are blocked by picrotoxin, suggesting the involvement of GABAA receptors (Franklin & Abbott, 1993). Differences between our study and other studies might be due to the different methods of scoring and defining intervals for formalin test. Besides, we should not forget the role of age, weight, and genetics of the animals used in different studies. Similar to our results, effective inhibitory mechanisms of pain in this period has been reported in some earlier studies (Henry et al., 1999).

Our results revealed that swimming in different heights of water had different effects on the nociception observed in various phases of the formalin test. Naloxone diminishes nociceptive scores in phase 1, interphase, and phase 2 compared to the control group. These findings suggest that opioid might be involved in swim stress-induced modulation of pain, and different heights of water employ different inhibitory mechanisms.

## References

[B1] Abbott F. V. Franklin K. B. Westbrook R. F. (1995). The formalin test: Scoring properties of the first and second phases of the pain response in rats. Pain, 60(1), 91–102. [DOI:10.1016/0304-3959(94)00095-V] 7715946

[B2] Akil H. Young E. Walker J. M. Watson S. J. (1986). The many possible roles of opioids and related peptides in stress-induced analgesia. Annals of the New York Academy of Sciences, 467(1), 140–53. [DOI:10.1111/j.1749-6632.1986.tb14625.x] 2942087

[B3] Altier N. Stewart J. (1999). The tachykinin NK-1 receptor antagonist, RP-67580, infused into the ventral tegmental area prevents stress-induced analgesia in the formalin test. Physiology & Behavior, 66(4), 717–21. [DOI:10.1016/S0031-9384(98)00246-7] 10386919

[B4] Amit Z. Galina Z. H. (1986). Stress-induced analgesia: Adaptive pain suppression. Physiological Reviews, 66(4), 1091–120. [DOI:10.1152/physrev.1986.66.4.1091] [PMID] 2876446

[B5] Azhdari-Zarmehri H. Erami E. Ghasemi E. Salmani M. E. (2012). [Chronic heterogeneous sequential stress increases formalin-induced nociceptive behaviour in male rats (Persian)]. Physiology and Pharmacology, 16(4), 371–9.

[B6] Azhdari-Zarmehri H. Mohammad-Zadeh M. Feridoni M. Nazeri M. (2014). Termination of nociceptive bahaviour at the end of phase 2 of formalin test is attributable to endogenous inhibitory mechanisms, but not by opioid receptors activation. Basic and Clinical Neuroscience, 5(1), 48–54. [PMID] [PMCID] 25436084 PMC4202598

[B7] Bodnar R. J. Kelly D. D. Brutus M. Glusman M. (1980). Stress-induced analgesia: Neural and hormonal determinants. Neuroscience & Biobehavioral Reviews, 4(1), 87–100. [DOI:10.1016/0149-7634(80)90028-7] 6995874

[B8] Butler R. K. Finn D. P. (2009). Stress-induced analgesia. Progress in Neurobiology, 88(3), 184–202. [DOI:10.1016/j.pneurobio.2009.04.003] [PMID] 19393288

[B9] Dubuisson D. Dennis S. G. (1977). The formalin test: A quantitative study of the analgesic effects of morphine, meperidine, and brain stem stimulation in rats and cats. Pain, 4, 161–74. [DOI:10.1016/0304-3959(77)90130-0] 564014

[B10] Fanselow M. S. Calcagnetti D. J. Helmstetter F. J. (1989). Role of mu and kappa opioid receptors in conditional fear-induced analgesia: The antagonistic actions of norbinaltorphimine and the cyclic somatostatin octapeptide, Cys2Tyr3Orn5Pen7-amide. Journal of Pharmacology and Experimental Therapeutics, 250(3), 825–30. 2570868

[B11] Franklin K. B. J. Abbott F. V. (1993). Pentobarbital, diazepam, and ethanol abolish the interphase diminution of pain in the formalin test: evidence for pain modulation by GABAA receptors. Pharmacology Biochemistry and Behavior, 46(3), 661–66. [DOI:10.1016/0091-3057(93)90558-B] 8278443

[B12] Gaumond I. Arsenault P. Marchand S. (2002). The role of sex hormones on formalin-induced nociceptive responses. Brain Research, 958(1), 139–45. [DOI:10.1016/S0006-8993(02)03661-2] 12468038

[B13] Gaumond I. Arsenault P. Marchand S. (2005). Specificity of female and male sex hormones on excitatory and inhibitory phases of formalin-induced nociceptive responses. Brain Research, 1052(1), 105–11. [DOI:10.1016/j.brainres.2005.06.011] [PMID] 16005855

[B14] Gaumond I. Spooner M. F. Marchand S. (2007). Sex differences in opioid-mediated pain inhibitory mechanisms during the interphase in the formalin test. Neuroscience, 146(1), 366–74. [DOI:10.1016/j.neuroscience.2007.01.002] [PMID] 17306464

[B15] Guillemin R. Vargo T. Rossier J. Minick S. Ling N. Rivier C. (1977). Beta-Endorphin and adrenocorticotropin are selected concomitantly by the pituitary gland. Science, 197(4311), 1367–9. [DOI:10.1126/science.197601] [PMID] 197601

[B16] Henry J. L. Yashpal K. Pitcher G. M. Coderre T. J. (1999). Physiological evidence that the ‘interphase’ in the formalin test is due to active inhibition. Pain, 82(1), 57–63. [DOI:10.1016/S0304-3959(99)00033-0] 10422660

[B17] Hopkins E. Spinella M. Pavlovic Z. W. Bodnar R. J. (1998). Alterations in swim stress-induced analgesia and hypothermia following serotonergic or NMDA antagonists in the rostral ventromedial medulla of rats. Physiology & Behavior, 64(3), 219–25. [DOI:10.1016/S0031-9384(98)00055-9] 9748086

[B18] Łapo I. B. Konarzewski M. Sadowski B. (2003). Effect of cold acclimation and repeated swimming on opioid and nonopioid swim stress-induced analgesia in selectively bred mice. Physiology & Behavior, 78(3), 345–50. [DOI:10.1016/S0031-9384(02)01004-1] 12676268

[B19] Madden J. Akil H. Patrick R. L. Barchas J. D. (1977). Stress-induced parallel changes in central opioid levels and pain responsiveness in the rat. Nature, 265(5592), 358–60. [DOI:10.1038/265358a0] [PMID] 189213

[B20] Mogil J. S. Sternberg W. F. Kest B. Marek P. Liebeskind J. C. (1993). Sex differences in the antagonism of swim stress-induced analgesia: effects of gonadectomy and estrogen replacement. Pain, 53(1), 17–25. [DOI:10.1016/0304-3959(93)90050-Y] 8316385

[B21] Oliverio A. Castellano C. (1982). Classical conditioning of stress-induced analgesia. Physiology & Behavior, 29(1), 171–2. [DOI:10.1016/0031-9384(82)90384-5] 7122728

[B22] Sofiabad M. Heidari N. Ghasemi E. Esmaeili M. H. Haghdoost-Yazdi H. (2011). [Assesment of orexin receptor 1 in stress attenuated nociceptive behaviours in formalin test (Persian)]. Physiology and Pharmacology, 15(3), 395–402.

[B23] Watkins L. R. Mayer D. J. (1986). Multiple Endogenous opiate and non-opiate analgesia systems: Evidence of their existence and clinical implicationsa. Annals of the New York Academy of Sciences, 467(1), 273–99. [DOI:10.1111/j.1749-6632.1986.tb14635.x] 3014973

